# Palladated Cyclodextrin Nanosponge-Alginate Dual Bead as an Efficient Catalyst for Hydrogenation of Nitroarenes in Aqueous Solution

**DOI:** 10.3390/polym15153240

**Published:** 2023-07-29

**Authors:** Samahe Sadjadi, Abolfazl Heydari

**Affiliations:** 1 Gas Conversion Department, Faculty of Petrochemicals, Iran Polymer and Petrochemical Institute, P.O. Box 14975-112, Tehran 14977-13115, Iran; 2 Polymer Institute of the Slovak Academy of Sciences, Dúbravská cesta 9, 845 41 Bratislava, Slovakia; abolfazl.heydari@savba.sk

**Keywords:** cyclodextrin nanosponge, sodium alginate, hydrogenation, catalyst

## Abstract

In this study, we present a novel composite material consisting of β-cyclodextrin nanosponge and sodium alginate, used as a support for the immobilization of palladium (Pd) nanoparticles. The composite alginate-cyclodextrin nanosponge beads were prepared, taking advantage of the 3D polymeric network and β-cyclodextrin cavity of the nanosponge. These beads exhibited excellent encapsulation capabilities for hydrophobic substrates, allowing their transfer in aqueous media. The cyclodextrin nanosponge served as a stabilizer for Pd nanoparticles and facilitated phase transfer. Additionally, the sodium alginate bead contributed to the robustness of the structure and improved the recovery and recyclability of the composite material. Comparative studies with control catalysts confirmed the beneficial effect of incorporating cyclodextrin nanosponge within alginate beads, particularly for more hydrophobic substrates. Optimization of reaction conditions revealed that employing 0.03 g of catalyst per mmol of nitroarene at 45 °C resulted in the maximum yield within 90 min. Evaluation of the substrate scope demonstrated the hydrogenation capability of various substrates with different electronic properties under the developed protocol. Notably, the nitro group was selectively reduced in substrates featuring competing functionalities. Furthermore, the recyclability and stability of the composite catalyst were confirmed, making it a promising candidate for sustainable catalysis.

## 1. Introduction

With the growing environmental concerns encompassing global warming, water scarcity, ecosystem pollution, and species extinction, the need for sustainable development has become imperative [1,2,3]. In response, utilization of natural compounds, such as clays and carbohydrates [4,5,6], which are biodegradable, environmental friendly, and readily available, has emerged as a potential solution to address both environmental and economic challenges. In chemical synthesis, the substitution of organic and/or toxic reagents and catalysts with bio-based materials can contribute to the development of green and environmentally-friendly protocols [7,8]. Carbohydrates, including sodium alginate (Alg) and cyclodextrins (CDs), represent a class of bio-based compounds that have been extensively explored for the development of eco-friendly catalysts.

CDs are cone-shaped carbohydrates characterized by a hydrophobic cavity [9,10] and a hydrophilic outer surface, with cavity sizes varying according to the CD type [11,12]. These distinctive features make CDs promising candidates for various research areas, including catalysis and drug delivery [13,14,15,16,17,18]. The CD cavity functions as a molecular shuttle capable of encapsulating guest molecules of appropriate size and polarity and transferring them in aqueous solutions. This property is particularly valuable in the design of phase transfer catalysts. Furthermore, polymerization of CDs can yield insoluble bio-based polymers, making them even more suitable for the development of heterogeneous catalysts. Cyclodextrin nanosponges (CDNS) [19,20], prepared by cross-linking cyclodextrin monomers with crosslinking agents, such as dimethyl carbonate and carbonyl diimidazole, benefit from the cavities within CDs and the three-dimensional polymeric network [21,22,23,24,25,26,27]. This class of polymers has found wide range of applications [28] both in chemical and pharmaceutical applications [29]. Additionally, task-specific CDNS can be tailored by adjusting the synthesis route, monomer type, and cross-linking agent. Importantly, CDNS also serve as effective catalyst supports and stabilizers for nanoparticles (NPs) [30].

In addition to CDs, sodium alginate is a versatile polysaccharide with diverse applications in chemistry and pharmacy. Derived from algae, this linear bio-copolymer comprises β-D-mannuronate (M) and α-L-guluronate (G) units. Notably, sodium alginate exhibits the unique ability to form hydrogels in the presence of divalent cations, such as calcium ions [31,32]. These Alg beads can be dried, which possess robustness without the need for crosslinking, distinguishing them from other carbohydrate-based beads. While sodium alginate hydrogels/beads find significant utility in cell encapsulation and drug delivery, they have also gained prominence in catalysis [8,33] due to their hydrophilicity, affordability, and relative durability. Additionally, various composite hydrogels and beads based on Alg have been reported, serving as effective matrices for immobilizing or encapsulating catalytic species [33,34,35].

Given the advantageous properties of CDNS and Alg, this study aims to design and synthesize novel composite beads, Alg-CDNS, composed of CDNS as a bio-based supporting material. Subsequently, the prepared Alg-CDNS will be palladated through the wet-impregnation method and employed as a heterogeneous catalyst for the hydrogenation of nitroarenes in aqueous solution. In this study, the catalytic activity of Alg-CDNS beads towards reduction of nitroarenes was investigated. It is hypothesized that CDNS within the catalyst structure can serve as both a stabilizer for Pd NPs and a molecular shuttle, acting as a phase transfer agent. The presence of Alg beads is expected to confer robustness to the catalyst and enhance its recovery and recyclability. Furthermore, the functional groups present in both Alg and CDNS can participate in anchoring and stabilizing Pd NPs.

## 2. Materials and Methods

### 2.1. Materials

Palladium (II) acetate (Pd(OAc)_2_, 99.98%), β-cyclodextrin (β-CD, >97%), sodium borohydride (NaBH_4_, >98%), diphenyl carbonate (99%), toluene (99.8%), methanol (MeOH, 99.8%), sodium alginate, calcium chloride (CaCl_2_, >93%) were all purchased from Sigma-Adrich (St. Louis, MO, USA) and applied for the synthesis of the catalytic composite. Nitroarenes (provided from Sigma-Aldrich, Darmstadt, Germany) were utilized for examining the activity of the as-prepared catalyst in the hydrogenation reaction.

### 2.2. Apparatus

The following analyses were used to verify formation of the designed catalyst: Coupled Plasma (ICP) analysis (Vista-pro device), X-ray diffraction (XRD, Siemens, Munich, Germany, D5000 equipped with Cu Kα radiation), thermogravimetric analysis (TGA, METTLER TOLEDO, Columbus, OH, USA, under nitrogen atmosphere and heating rate of 10 °C·min^−1^), Scanning electron microscopy (SEM, VEGAII TESCAN device, Brno, Czech Republic, equipped with QX2, RONTEC energy dispersive X-ray analyzer), transmission electron microscopy (TEM, Phillips EM 208S microscope, Beaverton, USAat 100 kV) and Fourier transform infrared spectroscopy (FTIR, PERKIN-ELMER-Spectrum 65 apparatus using KBr pellet, Waltham, MA, USA).

### 2.3. Preparation of Palladated Alg-CDNS Composite Beads (Pd/Alg-CDNS)

CDNS was prepared through a facile and well-established procedure [36]. Typically, the crosslinking agent, diphenyl carbonate (0.85 g), was heated in a beaker to melt. Subsequently, β-CD powder (0.56 g) was gradually added to the melted diphenyl carbonate to form a white solid called CDNS. Stirring was maintained at 120 °C for 20 h, and then the obtained solid was collected and crushed into a fine powder. Since the resultant CDNS may contain impurities, such as phenol and unreacted β-CD, it was washed with acetone and distilled water several times. Furthermore, purification was carried out using Soxhlet extraction with ethanol for 5 h.

The CDNS that was prepared earlier was used for the synthesis of alginate composite beads, referred as Alg-CDNS. In this process, 1 g of sodium alginate was dissolved in deionized water and mixed with 1 g of CDNS under stirring conditions at ambient temperature for 2 h. The resulting homogeneous suspension was then transferred to a burette. The suspension in the burette was slowly dispensed into a CaCl_2_ solution (4.4 g in 200 mL of deionized water) to form small white beads. After allowing the beads to soak in the CaCl_2_ solution for 24 h, they were collected, rinsed with distilled water, and left to dry at ambient temperature overnight.

**Figure 1:**
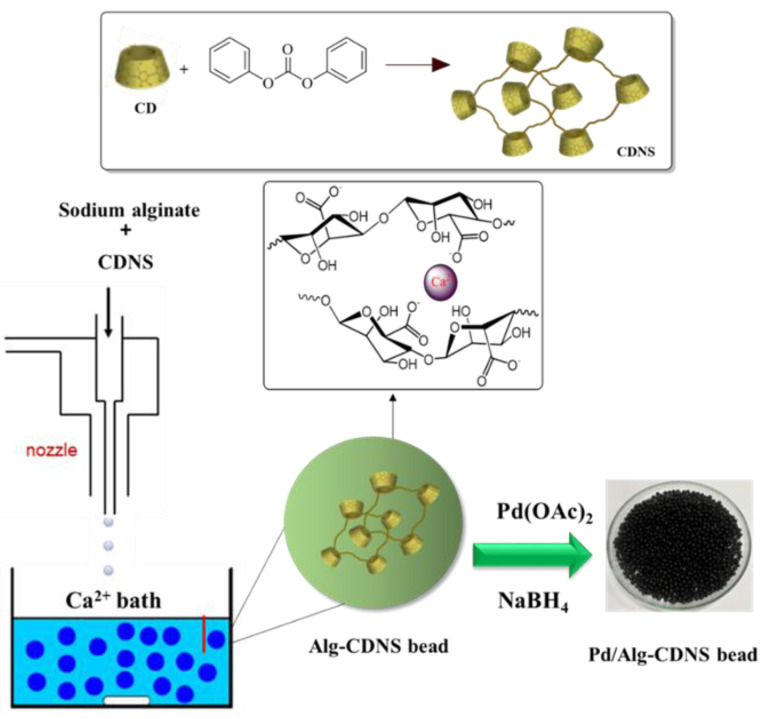
Pictorial Presentation of the Synthetic Route for Pd/Alg-CDNS.

Alg-CDNS beads were palladated using a known impregnation method [36]. In short, a solution of Pd(OAc)_2_ (0.03 g in 5 mL of toluene) was prepared and added drop by drop to the suspension of Alg-CDNS in toluene (1 g in 20 mL). The mixture was stirred for 4 h at ambient temperature. Simultaneously, a fresh NaBH_4_ solution (0.1 g in 15 mL of MeOH) was prepared and gradually added to the aforementioned mixture under an argon atmosphere. After mixing for 4 h at ambient temperature, the resulting black palladated beads, Pd/Alg-CDNS, were collected, washed with MeOH, and dried under vacuum overnight (see Figure 1). ICP analysis revealed that the loading of Pd NPs on the Pd/Alg-CDNS was 1.5 wt%.

### 2.4. Hydrogenation of Nitroarenes

To reduce nitrobenzene as a typical substrate, to their corresponding anilines, 6.15 g of nitrobenzene and 0.75 g of Pd/Alg-CDNS were mixed in 7 mL of deionized water under a hydrogen gas pressure of 1 bar. Stirring was continued at 45 °C, and the reaction progress was monitored using thin-layer chromatography (TLC). Notably, a hydrogen generator was used for providing hydrogen and it was transferred in the reaction vessel, which was a two-neck glass flask via hydrogen line and specially designed connecter, which was connected to one neck of the flask. The reaction vessel was sealed and for TLC, a tiny portion of the reaction mixture was obtained via syringe from the other neck of the flask without opening it. At the end of the reaction, the Pd/Alg-CDNS catalyst was collected, washed with MeOH several times, and dried at ambient temperature overnight for reuse. The resulting aniline was obtained by evaporating the solvent. The pure products were obtained via column chromatography. All of the products were known, so their formation was confirmed via comparing their boiling points with the authentic samples. It is worth noting that each experiment has been repeated for three times and the average of the results was used. The yield of the product was calculated using Equation (1), where, Mole (*N*) and (*A*) are the initial moles of nitrobenzene and mole of aniline respectively.

(1) $$Aniline\;yield\;\left(\%\right)=\frac{Mole\;\left(A\right)}{Mole\left(N\right)}\times 100\%$$

## 3. Results

### 3.1. Characterization of Pd/Alg-CDNS

**Figure 2:**
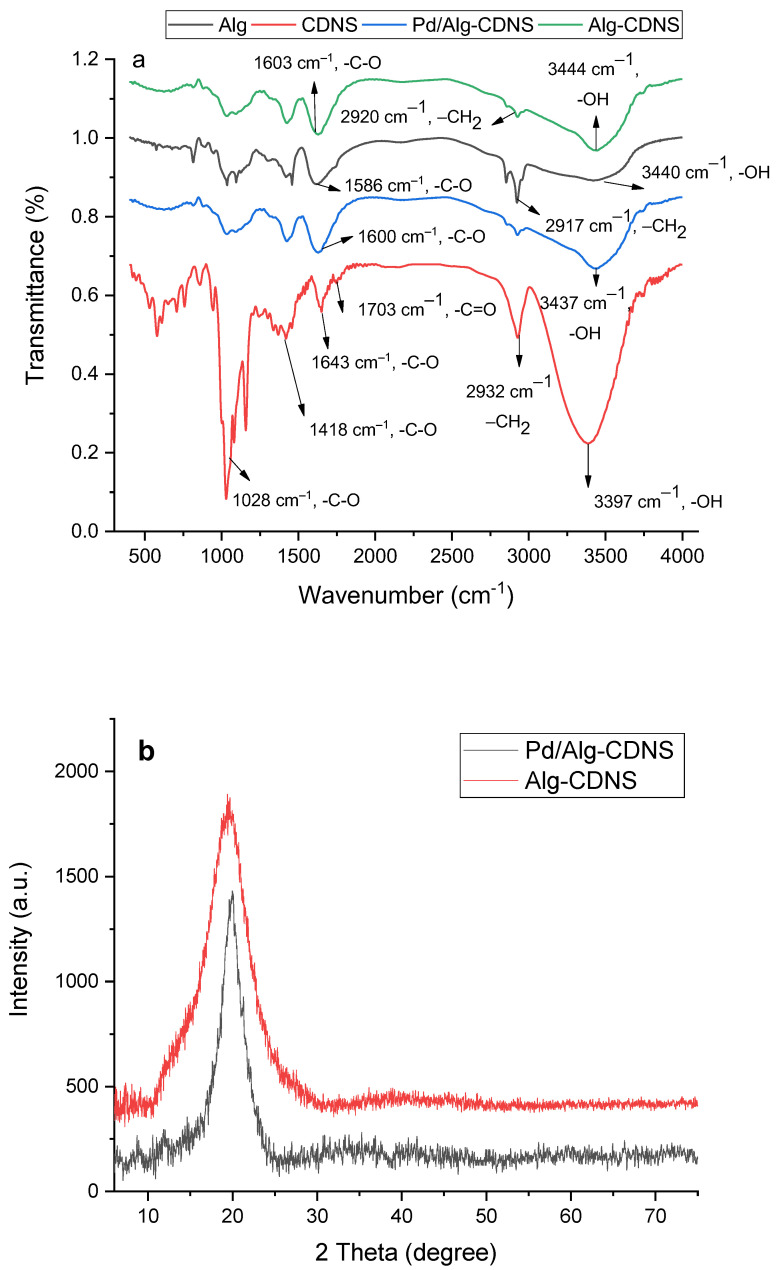

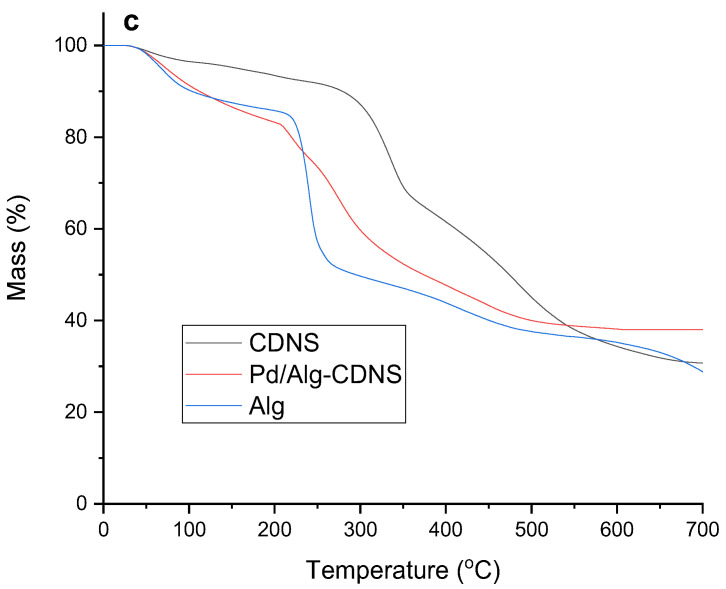
(**a**) FTIR spectra of CDNS, sodium alginate, and Pd/Alg-CDNS, (**b**) XRD patterns of the composite and Alg-CDNS (**c**) TGA curves of CDNS, sodium alginate, and Pd/Alg-CDNS.

The Pd/Alg-CDNS composite material was initially characterized using FTIR spectroscopy. To facilitate comparison, the FTIR spectra of the individual components of Pd/Alg-CDNS, namely CDNS and Alg, were also recorded and compared with the spectra of Alg-CDNS and Pd/Alg-CDNS (Figure 2a). In the FTIR spectrum of CDNS, the presence of the −CH functional group is evident from the absorbance bands observed at 3397 cm^−1^. Additionally, a band at 2932 cm^−1^ indicates the presence of −CH_2_ groups within the CDNS structure. Another noteworthy feature is a small absorbance band at 1703 cm^−1^, which is indicative of the −C=O functionality and confirms the successful crosslinking of CDs [30,37]. The FTIR spectrum of Alg closely resembles that of CDNS, with characteristic bands at 3450 cm^−1^, 2927 cm^−1^, and 1643 cm^−1^, corresponding to the −CH, −CH_2_, and −C=O functionalities, respectively [38,39]. Remarkably, the FTIR spectrum of Alg-CDNS, as a composite of CDNS and Alg, exhibits a remarkable resemblance to its individual components. It displays characteristic bands at 3430 cm^−1^ and 2937 cm^−1^, representing the −OH and −CH_2_ functionalities present within the catalyst’s structure, respectively. However, it should be noted that the characteristic band of the −C=O functionality overlaps with a broad band corresponding to −C–O functionality in the range of 1546–1755 cm^−1^. The FTIR spectrum of Pd/Alg-CDNS is very similar to that of Alg-CDNS and showed all of its characteristic bands. Noteworthy, the absorbance bands related to Pd NPs have been overlapped with those of Alg-CDNS. These FTIR spectroscopic findings provide valuable insights into the molecular composition and functional groups present in Pd/Alg-CDNS, supporting the successful integration of CDNS and Alg within the composite material.

The structure of Alg-CDNS and Pd/Alg-CDNS was examined using XRD analysis. Previous studies have reported that both Alg [8] and CDNS, synthesized through the melt method [36], exhibit an amorphous nature, displaying a broad peak at 2θ within the 15–30° range. In Figure 2b, the XRD patterns of both Alg-CDNS and Pd/Alg-CDNS display this distinctive peak, confirming the presence of the composite. Notably, the characteristic peaks expected for Pd nanoparticles (NPs), JCPDS Card No. 87-0639) [40], were not observed in the XRD pattern of Alg-CDNS, indicating the presence of finely dispersed Pd NPs [41]. This observation can be attributed to the high dispersion and small size of the Pd NPs within the composite.

TG analysis was conducted to further investigate the Pd/Alg-CDNS material prepared in this study. Figure 2c displays the TG curves of CDNS, Alg, and Pd/Alg-CDNS. The TG curve of CDNS exhibited a behavior consistent with previous findings [36], displaying weight loss corresponding to the evaporation of water and the decomposition of CDNS at temperatures of approximately 100 °C and 290 °C, respectively. Similarly, the TG curve of Alg revealed two distinct weight losses: the first peak observed at around 100 °C, attributed to the loss of water, and the second peak occurring at approximately 240 °C, corresponding to the decomposition of Alg. Notably, the TG analysis of Pd/Alg-CDNS displayed three discernible weight losses. The first weight loss occurred at around 100 °C, associated with the removal of water. The second weight loss, observed at 240 °C, was attributed to the decomposition of Alg. Finally, a third weight loss was detected at approximately 290 °C, indicative of CDNS decomposition. These results provide valuable insights into the thermal stability and decomposition behavior of Pd/Alg-CDNS, highlighting the presence of distinct weight loss stages associated with the individual components of the composite material.

**Figure 3:**
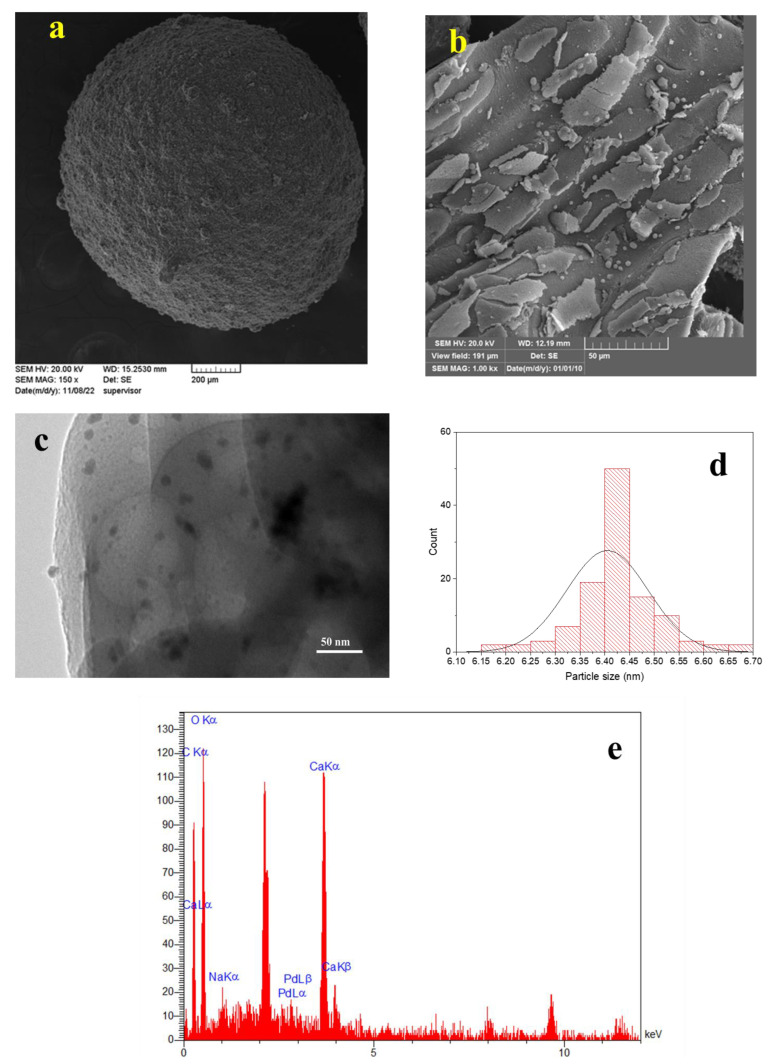
(**a**,**b**) SEM and (**c**) TEM images of Pd/Alg-CDNS, (**d**) particle size distribution curve of Pd NPs, and (**e**) EDS analysis of Pd/Alg-CDNS.

SEM images were obtained to examine the morphology of the Pd/Alg-CDNS composite. As shown in Figure 3a,b, the composite exhibited fine beads with an average diameter of 1.3 ± 0.5 mm and a rough surface. TEM analysis was further conducted to investigate the morphology of Pd/Alg-CDNS and measure the average particle size of the formed Pd NPs. As depicted in Figure 3c,d, Pd NPs with an average diameter of 6.4 ± 0.08 nm were uniformly dispersed on Alg-CDNS. However, some instances of Pd nanoparticle aggregation were also observed. EDS analysis of Pd/Alg-CDNS (Figure 3e) confirmed the presence of Pd, Ca, Na, C, and O atoms within the structure of the catalytic composite. The presence of C, O, and Na atoms was attributed to sodium alginate, while the presence of Ca atoms indicated interactions between Ca^2+^ and sodium alginate, visually evident from the formation of the beads. Notably, the C and O atoms were also associated with the CDNS structure. The detection of Pd atoms confirmed the successful impregnation of Pd NPs onto Alg-CDNS. These morphological and elemental characterizations provide valuable insights into the structure and composition of the Pd/Alg-CDNS composite, elucidating the uniform dispersion of Pd NPs on the support material.

### 3.2. Activity of Pd/Alg-CDNS in Hydrogenation of Nitroarenes

#### Combination Merit of Alg and CDNS

Considering the potential of CDNS as a capping agent for the NPs and its capability as a solid and heterogeneous phase transfer agent, which can encapsulate the guest molecules in the cavities of CD monomers and/or the cavities of the polymeric network, a novel composite of Alg and CDNS was prepared and applied as a bio-based support for the immobilization of Pd NPs. It was assumed that the functionalities on both Alg and CDNS could participate in the stabilization of Pd NPs. On the other hand, formation of beads of Alg-CDNS could improve the recovery and reuse of the composite. To verify these assumptions, a model reaction, hydrogenation of nitrobenzene in aqueous media, was selected and conducted in the presence of 0.02 g catalyst per 1 mmol of the substrate (nitrobenzene) at 40 °C. Furthermore, to elucidate the role of each component in the catalytic composite, two control catalysts, i.e., Pd/Alg and Pd/CDNS were also synthesized and their activity for the model reaction was studied under similar conditions, Table 1. As shown, Pd/Alg-CDNS composite could promote the model reaction to give aniline in 86% yield. Both Pd/Alg and Pd/CDNS showed good catalytic activity and led to the formation of aniline in 74 and 78%. Comparison of the activity of the three catalysts also approved that the activity of Pd/Alg and Pd/CDNS was lower compared to that of the composite, Table 1. These results indicated that the incorporation of CDNS in the alginate bead improved its catalytic activity. Comparison of the activity of Pd/Alg and Pd/CDNS for the hydrogenation of nitrobenzene in aqueous media approved their similar activity. This can be attributed to the nature of nitrobenzene as a substrate. More precisely, although nitrobenzene is a hydrophobic substrate, it is soluble in aqueous media. Hence, the role of CDNS as a phase transfer catalyst is not pronounced. To further approve CDNS role, a more steric and hydrophobic substrate, 1-nitronaphthalene, was selected as a model substrate and the activity of the so-called catalysts was investigated under the similar reaction conditions, Table 1. As summarized in Table 1, the activity of catalysts for hydrogenation of 1-nitronaphthalene followed the order of Pd/Alg-CDNS > Pd/CDNS > Pd/Alg. In fact, in the case of hydrophobic 1-nitronaphthalene that is less soluble in aqueous media, superior catalytic activity of Pd/CDNS compared to Pd/Alg is more obvious, confirming the role of CDNS as a phase transferring agent for performing the reaction in aqueous media.

### 3.3. Optimization of the Reaction Conditions

**Figure 4:**
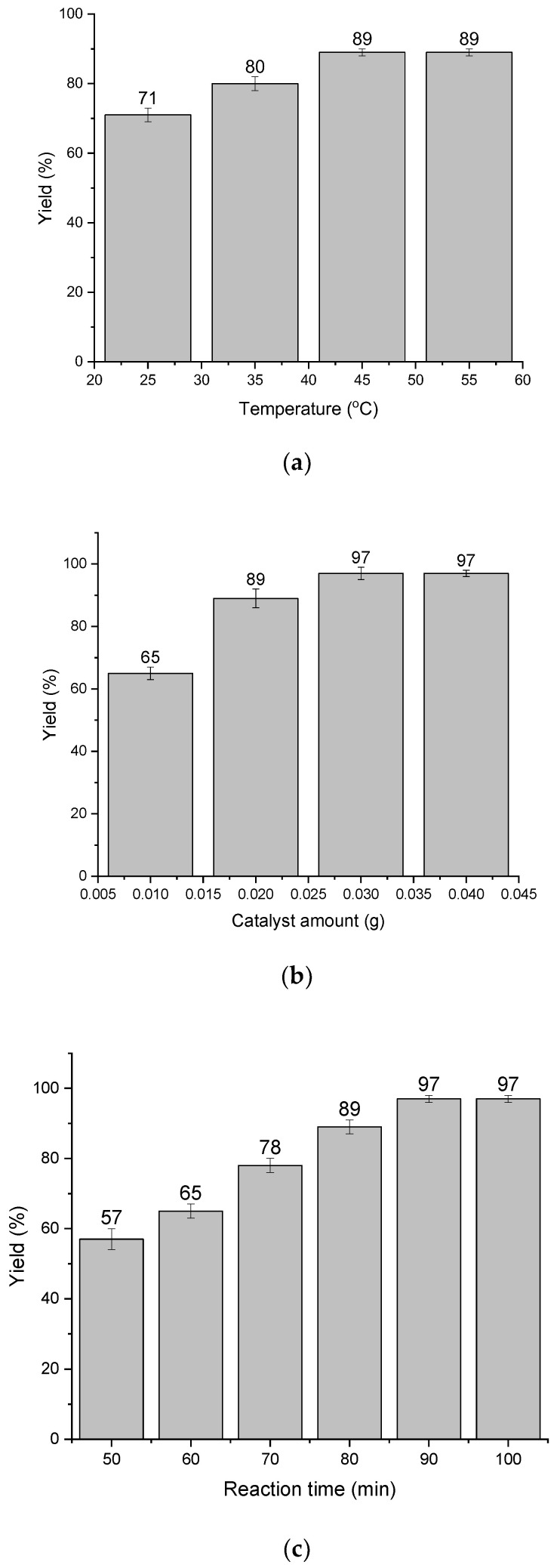
(**a**) The effect of the reaction temperature for the hydrogenation of nitrobenzene under hydrogen atmosphere (1 bar) using Pd/Alg-CDNS (0.02 g) in water. The reaction time is 90 min. (**b**) The effect of Pd/Alg-CDNS amount for the hydrogenation of nitrobenzene under hydrogen atmosphere (1 bar) at 45 °C in water. The reaction time is 90 min. (**c**) The effect of the reaction time for the hydrogenation of nitrobenzene under hydrogen atmosphere (1 bar) at 45 °C using Pd/Alg-CDNS (0.03 g) in water.

The initial tests demonstrated the high activity of Pd/Alg-CDNS for the hydrogenation of nitrobenzene. To maximize the yield of aniline, the reaction conditions, including reaction time, temperature, and Pd/Alg-CDNS amount, were optimized. The model reaction was first conducted in water with a fixed Pd/Alg-CDNS amount of 0.02 g under a hydrogen pressure of 1 bar at various temperatures ranging from 25 to 55 °C. The comparison of aniline yields (Figure 4a) clearly confirmed the influence of reaction temperature on the nitrobenzene hydrogenation yield, showing an increase in yield as the temperature was raised from 25 to 45 °C. However, further elevation of the temperature did not result in a significant increase in yield. Hence, the optimum reaction temperature was determined to be 45 °C, and subsequent experiments were conducted at this optimal temperature.

The influence of Pd/Alg-CDNS loading on the aniline yield was investigated by conducting the model reaction under a hydrogen atmosphere (1 bar) at 45 °C in water. The results, summarized in Figure 4b, demonstrated a consistent increase in aniline yield as the Pd/Alg-CDNS loading increased from 0.01 g to 0.03 g. The maximum yield of 97% was achieved at the optimal loading of 0.03 g per mmol of nitrobenzene. Notably, further increasing the catalyst loading did not lead to any significant improvement in the reaction yield, confirming that 0.03 g of Pd/Alg-CDNS was the optimum loading for this catalytic system.

To evaluate the impact of reaction time, the yield of nitrobenzene hydrogenation under a hydrogen atmosphere of 1 bar at 45 °C using Pd/Alg-CDNS (0.03 g) was determined at specific time intervals. As shown in Figure 4c, a linear relationship between reaction time and aniline yield was observed when extending the reaction from 50 to 90 min. However, no further improvement in yield was observed beyond a reaction time of 100 min. Based on these findings, the optimal reaction conditions were identified as using 0.03 g of Pd/Alg-CDNS per mmol of nitrobenzene at 45 °C for a reaction time of 90 min.

### 3.4. Generality

According to the results for the model reaction, it can be deduced that Pd/Alg-CDNS efficiently catalyzed hydrogenation of nitrobenzene to aniline. It is also important to elucidate whether Pd/Alg-CDNS can promote reaction of other nitroarenes with different features. To this purpose, several nitroarenes with electron-donating and electron-withdrawing functional groups were examined as substrates, Table 2 entries 1–8. As listed in Table 2, Pd/Alg-CDNS is an efficient catalyst for hydrogenizing various nitroarenes with different electronic features. More precisely, the electronic feature of the substrate slightly affected the reaction and the substrates with electron-withdrawing groups that are more active substrates led to the products with slightly higher yields. Considering the results presented in Table 2, entry 9, it is concluded that hydrogenation of steric substrates is less efficient than small ones. Moreover, the data displayed in Table 2, entry 10 indicates high selectivity of Pd/Alg-CDNS towards reduction of −NO_2_ functionality. More accurately, a substrate with both −NO_2_ group and Keto functionality (4-nitroacetophenone) was reduced selectively to give 4-aminoacetophenone as sole product.

### 3.5. Recyclability of Pd/Alg-CDNS

**Figure 5:**
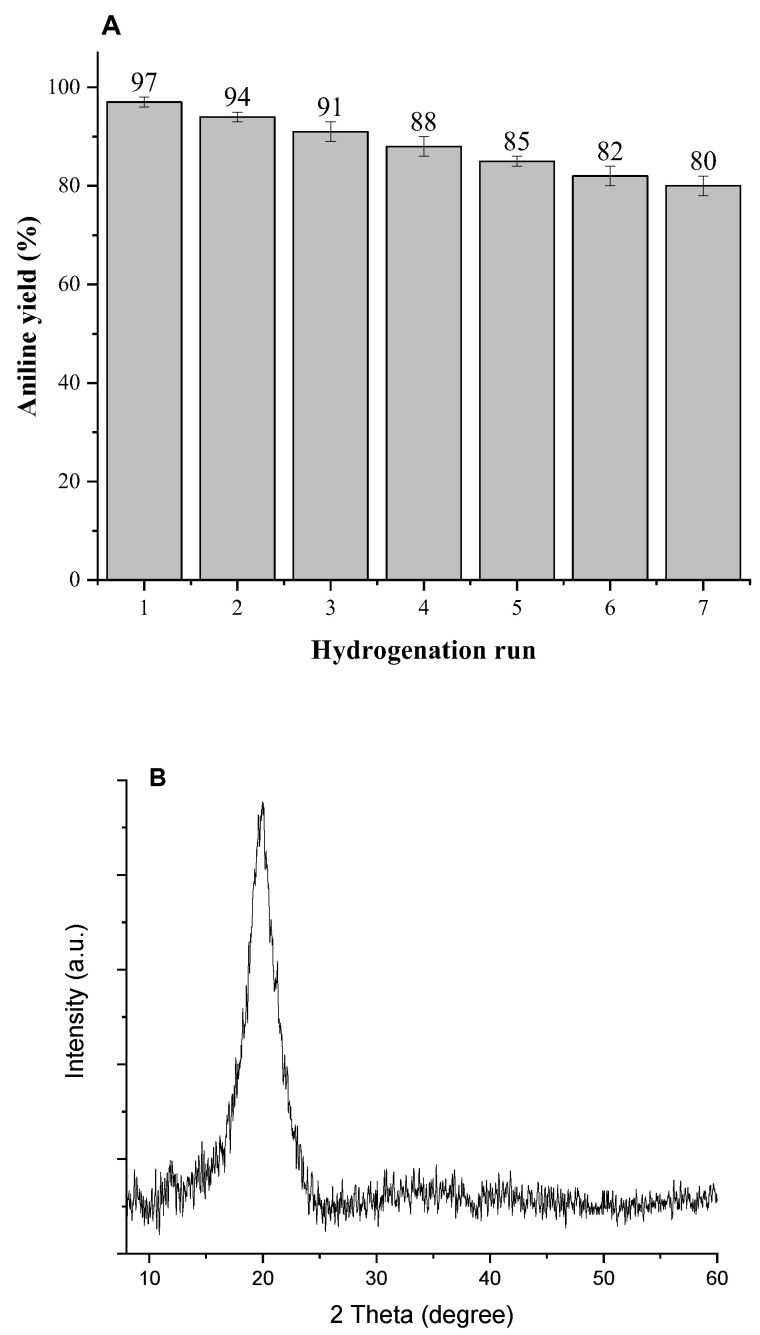

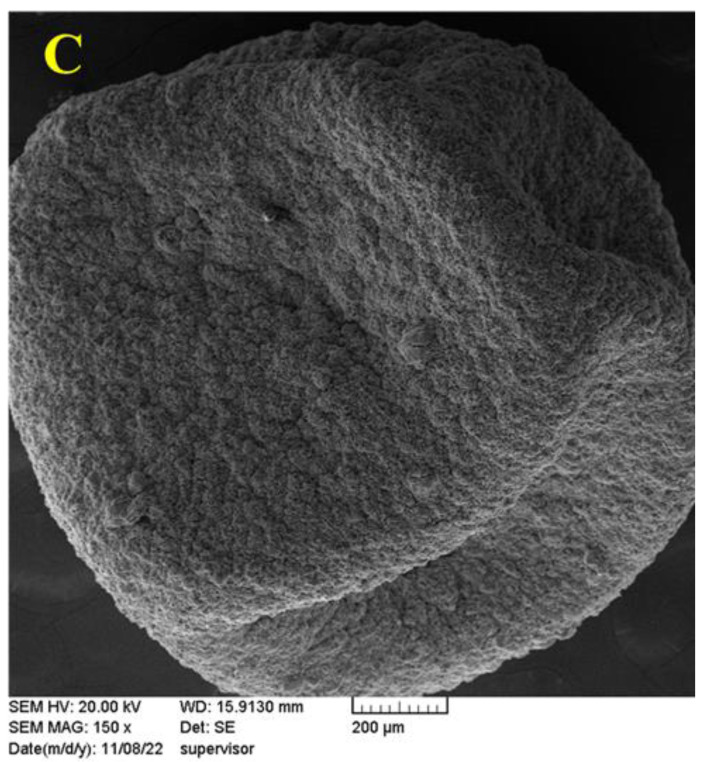
(**A**) yield of aniline in hydrogenation of nitrobenzene under the optimal reaction conditions. (**B**) XRD pattern of the reused Pd/Alg-CDNS after the seventh run. (**C**) SEM image of the reused catalyst after the last run of the reaction.

To measure the recyclability of Pd/Alg-CDNS, hydrogenation of nitrobenzene to aniline under the optimized reaction conditions was considered as a model reaction. At the end of the reaction, Pd/Alg-CDNS beads were readily separated from the reaction mixture by conventional filtration. To recover Pd/Alg-CDNS, it was rinsed with MeOH repeatedly and then dried at ambient temperature overnight. The second run of the reaction was conducted using the recovered Pd/Alg-CDNS under the same conditions and the yield of aniline was measured and compared with the previous run. Gratifyingly, comparison of yields of aniline for seven runs, Figure 5, confirmed insignificant loss of activity and high recyclability of Pd/Alg-CDNS. Morphological study of the reused catalyst was also performed by recording the SEM image of Pd/Alg-CDNS obtained from the last run of recycling. As shown in Figure 5C, the spherical morphology of the fresh beads could be slightly affected upon recycling and deformed beads were observed after several runs of recycling. However, similar to fresh beads, the surface of the reused beads was rough.

The leaching of metallic species is a crucial issue that can significantly affect the recyclability of metallic catalysts. Therefore, the Pd/Alg-CDNS catalyst recovered after the seventh run of the reaction was analyzed using ICP analysis. For ICP analysis, the recovered catalyst was analyzed with ICP. Encouragingly, the results revealed insignificant Pd leaching after each reaction run. More precisely, Pd loading in fresh catalyst (1.5 wt%) reached to 1.45, 1.4, 1.34. 1.3, 1.26, 1.2 wt% after second, third, fourth, fifth, sixth and seventh reaction run respectively. This indicates that the Alg-CDNS bead, which possesses various functionalities in its structure, is a suitable support for stabilizing Pd nanoparticles (NPs). To further elucidate the structure of the reused catalyst, the Pd/Alg-CDNS sample collected after the seventh run of the reaction was analyzed using XRD. Figure 5 shows that the XRD pattern of the reused Pd/Alg-CDNS is identical to that of the fresh catalyst, displaying a single characteristic peak at 2 theta in the range of 10–27°. This observation implies that Pd/Alg-CDNS is a stable composite, retaining its structural integrity even after multiple reaction cycles.

### 3.6. Comparative Study

Hydrogenation of nitroarenes plays a crucial role in the synthesis of various valuable chemicals, leading to a significant focus on catalyst development for this reaction. To compare the activity of Pd/Alg-CDNS with previous reports, several catalysts used for nitrobenzene hydrogenation were randomly selected, and their activity under reported conditions was compared in Table 3. While hydrogen gas is commonly employed as a reducing agent, some reports have utilized NaBH_4_, a chemical reagent. Although NaBH_4_ demonstrated good results, it is considered less environmentally benign compared to hydrogen gas. Moreover, protocols utilizing high pressure hydrogen gas were deemed unattractive due to safety concerns. In some cases, organic solvents such as THF were used, which are less desirable compared to aqueous media. Furthermore, certain catalyst designs involved complicated synthetic procedures (see Table 3, entries 3 and 10). In the case of entry 9 in Table 3, the use of a co-catalyst was necessary to achieve high reaction yields. Considering the results compiled in Table 3, it can be concluded that Pd/Alg-CDNS, as a bio-based catalyst prepared through a relatively straightforward procedure, exhibits efficient catalytic activity comparable to or even superior to some previous catalysts.

## 4. Conclusions

We have successfully designed and prepared novel composite alginate beads by incorporating CDNS, acting as a molecular shuttle, during the beads preparation process. The composite beads served as an effective support for stabilizing Pd NPs, benefiting from the combined functionalities of alginate and CDNS, which enhanced Pd anchoring. The resulting composite, Pd/Alg-CDNS, exhibited excellent catalytic activity in the hydrogenation of nitroarenes in aqueous media. Through optimization of reaction parameters, we achieved the highest product yields using 0.03 g of Pd/Alg-CDNS per mmol of nitrobenzene at 45 °C.

Significantly, comparative studies involving control catalysts, Pd/Alg and Pd/CDNS, confirmed that the incorporation of CDNS in the alginate beads led to improved catalyst performance, particularly for sterically demanding substrates like 1-nitronaphthalene. Encouragingly, Pd/Alg-CDNS demonstrated remarkable recyclability, retaining its activity for up to seven consecutive runs without a significant decrease. Importantly, this protocol can be extended to other nitroarenes with diverse electronic properties. Notably, Pd/Alg-CDNS exhibited selectivity towards nitro group reduction.

## Figures and Tables

**Table 1:** Performance comparison of control catalysts for nitroarene hydrogenation.

Entry	Catalyst	Yield (%) ^a^	Yield (%) ^b^
1	Pd/Alg-CDNS	86	75
2	Pd/Alg	74	42
3	Pd/CDNS	78	56

^a^ Reaction conditions: nitrobenzene (1 mmol), catalyst (0.02 g) in H_2_O at 40 °C under H_2_ gas (1 bar). ^b^ Reaction conditions: 1-nitronaphthalene (1 mmol), catalyst (0.02 g) in H_2_O at 40 °C under H_2_ gas (1 bar).

**Table 2:** The catalytic activity of Pd/Alg-CDNS in the hydrogenation of various nitroarenes.

Entry	Substrate	Product	Yield (%)
1	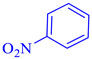	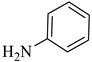	97
2	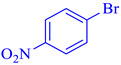	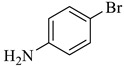	97
3	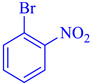	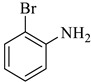	95
4	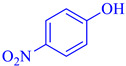	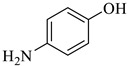	90
5	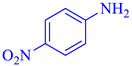	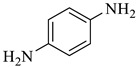	89
6	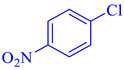	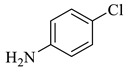	96
7	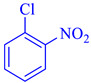	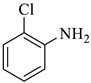	94
8	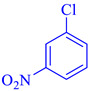	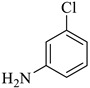	91
9	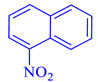	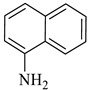	88
10	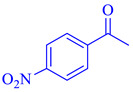	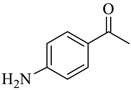	91

**Table 3:** Comparison of Pd/Alg-CDNS with randomly selected catalysts for hydrogenation of nitrobenzene.

Entry	Catalyst	Temp. (°C)	Reducing Agent	Time (min)	Solvent	Yield (%)	Ref.
1	Pd/Alg-CDNS	45	H_2_/1 bar	90	H_2_O	97	This work
2	PdNP (0.5%)/Al_2_O_3_	r.t.	H_2_/1 atm	180	THF	100	[42]
3	Pd@CS-CD-MGQDs ^a^ (0.5 mol%)	50	H_2_/1 atm	60	H_2_O	97	[43]
4	PdCu/graphene (2 mol% Pd)	50	NaBH_4_	90	H_2_O/EtOH	95	[44]
5	Pd/graphene	50	NaBH_4_	90	H_2_O/EtOH	91	[44]
6	Pd@Hal-biochar ^b^ (0.03 mol%)	r.t.	H_2_/1 bar	60	H_2_O	75	[45]
7	APSNP ^c^ (1 mol%)	r.t.	H_2_/20 atm	120	EtOH	100	[46]
8	Pd/PPh_3_@FDU-12 (8.33 × 10^−4^ mmol Pd)	40	H_2_/10 bar	60	EtOH	>99	[47]
9	Pd@Hal-Hydrogel + cyclodextrin (2 wt%)	50	H_2_/1 bar	120	H_2_O	95	[48]
10	Pd@Hal-TCT-Met	65	H_2_/1 bar	75	H_2_O	100	[49]
11	Pd@Hal/di-urea ^d^	50	H_2_/1 bar	60	H_2_O	100	[50]
12	Pd@Hal-CCD ^e^	r.t.	H_2_/1 bar	90	H_2_O	100	[51]
13	PdCu/C (2 mol% Pd)	50	NaBH_4_	90	H_2_O/EtOH	85	[44]

^a^ Pd on composite of magnetic graphene dots and CD decorated chitosan. ^b^ Composite of clay and char. ^c^ Activated palladium sucrose nanoparticles. ^d^ Ligand decorated clay. ^e^ Halloysite decorated with CD derived carbon sphere.

## Data Availability

Not applicable.
